# Endocardial-Myocardial Interactions During Early Cardiac Differentiation and Trabeculation

**DOI:** 10.3389/fcvm.2022.857581

**Published:** 2022-05-04

**Authors:** Xianghu Qu, Cristina Harmelink, H. Scott Baldwin

**Affiliations:** ^1^Department of Pediatrics (Cardiology), Vanderbilt University Medical Center, Nashville, TN, United States; ^2^Department of Cell and Development Biology, Vanderbilt University, Nashville, TN, United States

**Keywords:** endocardial cell, cardiac development, endocardial-myocardial interactions, myocardial trabeculation, cardiomyocyte, endocardial growth

## Abstract

Throughout the continuum of heart formation, myocardial growth and differentiation occurs in concert with the development of a specialized population of endothelial cells lining the cardiac lumen, the endocardium. Once the endocardial cells are specified, they are in close juxtaposition to the cardiomyocytes, which facilitates communication between the two cell types that has been proven to be critical for both early cardiac development and later myocardial function. Endocardial cues orchestrate cardiomyocyte proliferation, survival, and organization. Additionally, the endocardium enables oxygenated blood to reach the cardiomyocytes. Cardiomyocytes, in turn, secrete factors that promote endocardial growth and function. As misregulation of this delicate and complex endocardial-myocardial interplay can result in congenital heart defects, further delineation of underlying genetic and molecular factors involved in cardiac paracrine signaling will be vital in the development of therapies to promote cardiac homeostasis and regeneration. Herein, we highlight the latest research that has advanced the elucidation of endocardial-myocardial interactions in early cardiac morphogenesis, including endocardial and myocardial crosstalk necessary for cellular differentiation and tissue remodeling during trabeculation, as well as signaling critical for endocardial growth during trabeculation.

## Introduction

The process of heart development can be broken down into a handful of basic steps according to major morphogenic landmarks. First is the formation of the moon-shaped cardiac crescent, followed by coalescence to create the linear heart tube, which then loops and subsequently undergoes formation of discrete cardiac chambers, myocardial trabeculation, and valvulo-septa morphogenesis (1, 2). While endocardium-myocardium crosstalk appears to be involved in all these developmental milestones, it is especially important during trabeculation and valve formation (1, 3–5). Improper endocardial-myocardial communication leads to a failure of cardiac growth and embryonic lethality or congenital heart diseases (6–10). This review attempts to encapsulate what is currently known about the unique, reciprocal communication between endocardial cells and cardiomyocytes during early cardiac development, including endocardial and myocardial crosstalk during their differentiation, myocardial trabeculation, and endocardial growth during trabeculation. The endocardial and myocardial signaling during cardiac endocardial-to-mesenchymal transformation and endocardial cushion formation, a well-studied process, has been comprehensively reviewed elsewhere (5, 11–13) and will not be discussed here. Similarly, the potential contribution of the endocardium to coronary vascular development and extracardiac organogenesis has been recently summarized (10) and is beyond the scope of this review.

## Origins of the Endocardium and Relationship With Myocardium

During mouse embryogenesis, the endocardium is initially morphologically identifiable at the one- to two- somite stage as a “proendocardium layer” residing between the myocardial and endodermal layers (14). Molecularly, early endocardial cells can be distinguished by endocardial-specific expression of the transcription factor *Nfatc1* (15). Recently the endocardial-specific molecular signature that differentiates them from other endothelial cells has been elaborated upon using single cell RNAseq analyses. This distinctive signature includes expression of markers such as natriuretic peptide receptor 3 (16), cytokine-like protein 1 (17), MEIS2, HAPLN1, FOXC1, LEPR, and TMEM100 (18) within the endocardium.

The embryonic origins of the myocardium have received much attention and is described in many excellent reviews (2, 19, 20). Extensive studies have primarily used avian models because of the easily accessible embryo. These studies have revealed that, within the heart-forming region, mesodermal differentiation into myocardium is governed by positive signals (BMP, FGF, TGF-β and Hedgehog pathways) and inhibitory signals including Wnts, Chordin, and Noggin (2, 21). However, the timing of endocardial specification, as well as the origins of endocardial vs. myocardial lineages, remain controversial (22, 23). For example, fate-mapping data, largely gleaned from chick and zebrafish models, provide evidence for *separate* origins. In these studies, cells were retrovirally tagged, which allowed cells to be tracked as they migrated from the primitive streak to the cardiac crescent. Results from these zebrafish studies demonstrated a separation of endocardial- and myocardial-destined cells during early gastrulation, with a progenitor cell only differentiating into one cell type or the other (24–26). Similarly, results from studies using chicken embryos provide data that myocardial and endocardial progenitors are independent from one another at the primitive streak stage or perhaps even earlier (27–30). Overall, these data support a model wherein pre-specification of mesodermal cells within the primitive streak dictates either an endocardial or a myocardial fate.

More recently, studies utilizing mouse models, cell lineage-mapping, and embryonic stem cells (ESCs) have shown that endocardial and myocardial precursors have common spatial and molecular characteristics, which ultimately supports the idea that they are derivatives of a common multipotent cell residing within the cardiac mesoderm (22, 26). For instance, lineage tracing in mice revealed a multipotent population of precardiac progenitors in the late primitive streak that express vascular endothelial growth factor receptor 2 (Vegfr2, or Flk1) and were capable of generating cardiomyocytes and endothelial cells (31). Substantiating that data are studies using Cre lines driven by cardiogenic promoters (Nkx2-5-Cre, Isl1-Cre, and the Mef2C-enhancer-Cre) to trace cell lineages, all of which led to the labeling of both endocardial cells and cardiomyocytes (32–34). Additionally, embryonic murine Isl1^+^ cardiac progenitors proved to be multipotent *in vivo* and *in vitro*, giving rise to endocardial, myocardial, and smooth muscle cells (35). Another link between endocardial and myocardial specification comes from studies showing that the cardiac transcription factor NK2 homeobox 5 (Nkx2-5) promotes expression of the ETS-related transcription factor gene *Etv2* (*Er71* or *Etsrp71*) (36), which is necessary for endocardial cell specification (discussed below) (23, 36, 37). Removal of Etv2 results in myogenic differentiation of cells that would otherwise have become endocardium (38). Finally, experiments using ESC differentiation not only validate that a common multipotent progenitor cell gives rise to myocardial, smooth muscle, and endocardial cells, but provide evidence that endocardial cell progenitors are derived from a distinct lineage when compared to other haematopoietically-derived vascular endothelial cells (31, 35, 39–41). Together these data illustrate a shared origin story beginning in a multipotent myocardial-endocardial progenitor cell during the late primitive streak stages.

How to reconcile the above, apparently opposing sets of data? The answer may lie with a prolonged developmental plasticity of the progenitor cells. For instance, as briefly mentioned above, endocardial cells can be reprogrammed into muscle cell lineages, including myocardium, through loss of Etsrp/Etv2 function (38, 42). Notably, using Mesp1^*Cre*^ to permanently label the earliest cardiovascular progenitor population within the early primitive streak stage (E6.25), Mesp1+ cells within the first heart field heterogeneously expressed endocardial and myocardial markers in an “either or” fashion. Interestingly, this changed during the late primitive streak stage (E7.25), when the Mesp1-expressing lineage in the second heart field more inclusively expressed endocardial and myocardial markers (29, 43, 44). These data from zebrafish and mammalian studies allow an integration of the above-mentioned, seemingly at-odds research by setting up a plausible scenario wherein multipotent endocardial-myocardial progenitors remain developmentally pliable longer than anticipated (23).

## Endocardial-Myocardial Crosstalk During Their Differentiation

**Figure 1:**
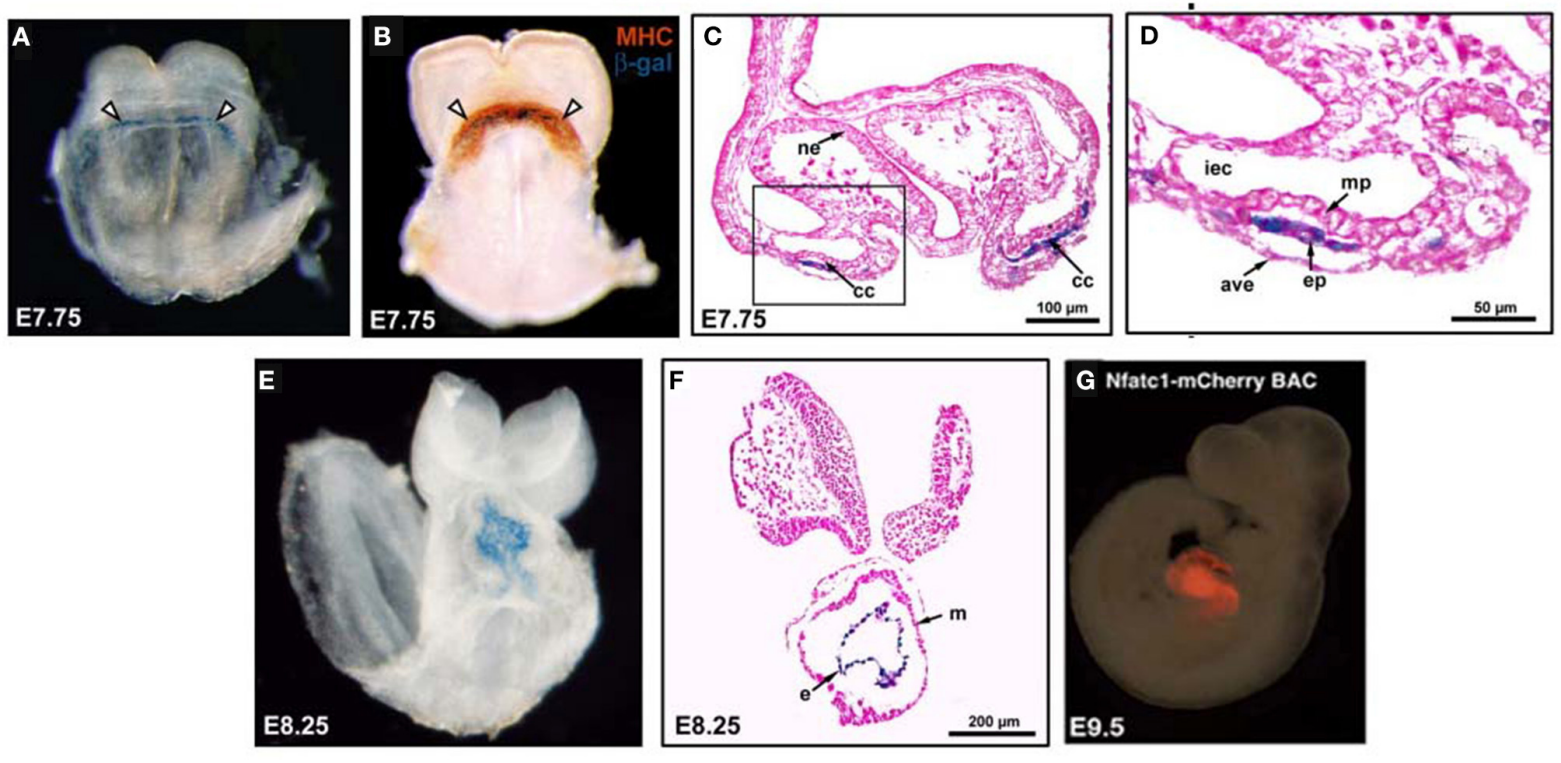
*The endocardium and myocardium are intimately associated during early differentiation*. **(A)** An NFATc1-nuc-LacZ embryo, E7.75, stained with X-Gal. Nuclear expression of β-gal (blue) within the endocardium of the cardiac crescent (white arrowheads). **(B)** An E7.75 NFATc1-nuc-LacZ embryo stained with myosin heavy chain (MHC; reddish-brown) to identify the myocardium and co-stained with X-Gal to mark β-gal+ (blue) endocardium within the cardiac crescent (white arrowheads). **(C,D)** E7.75 embryo coronal sections revealing blue β-gal+ endocardial cells between the myocardial precursors and the anterior endoderm. mp, myocardial precursors; ep, endocardial precursor; ne, neuroectoderm; cc, cardiac crescent; ave, anterior visceral endoderm; iec, intraembryonic coelom. **(E)** X-Gal stained E8.25 NFATC1-nuc-LacZ transgenic embryo with blue β-gal expression confined to the endocardial layer of the linear heart tube. **(F)** Coronal sections of E8.25 NFATc1-nuc-LacZ embryo revealing blue β-gal expression specifically and exclusively in the endocardium. **(G)** Whole-mount immunofluorescence of an NFATc1-mCherry (BAC) E9.5 embryo confirms endocardium-specific mCherry expression [adapted from Misfeldt et al. (40) and Saint-Jean et al. (45)].

It is likely that there are interactions between the myocardial and endocardial cells from the earliest development stages, given that the myocardium develops concurrently with the endocardial layer and that the two populations of cells are intimately associated (Figure 1). Yet the paucity of available models to study myocardial differentiation without contributions from an endocardial population has made it difficult to further delineate the endocardial-myocardial signaling. Saint-Jean et al. (45) recently developed a novel *in vitro* mouse ESC system to determine how myocardial differentiation is dependent on the endocardium, focusing on the initial stages of cardiogenesis. Using regulatory elements of the endocardial-specific marker, Nfatc1, they created a mouse stem cell line that expresses the diphtheria toxin receptor (Nfatc1-DTR) only within differentiated endocardial cells. During early stages of *in vitro* differentiation, the *NFATc1-DTR* mouse embryoid bodies were treated with diphtheria toxin to ablate endocardial cells, while other endothelial populations remained intact. Loss of the endocardial cells was detrimental to cardiomyocyte function and differentiation, with less beating embryoid bodies and reduced expression of genes necessary for early and late myocardial differentiation. Bmp2 was found to partially rescue the myocardial function and gene expression, suggesting that initial stages of myocardial differentiation are mediated by endocardially-derived Bmp2. Supporting these data, Pasquier et al. (46) found that endothelial cells augmented cardiomyocyte maturation when co-cultured with human ESCs.

In turn, early endocardial differentiation is dependent on myocardial paracrine signaling. One recent study used differentiation of human pluripotent stem cell (hPSC)-derived cardiovascular mesoderm to investigate endocardial differentiation. In this model, cells were treated with BMP10 (a growth factor secreted by cardiomyocytes), NRG1 (an endocardial-expressed growth factor), or inhibitors including siRNA against *BMP10* and *NRG1* (18). They found that hPSC-derived endocardial cells require BMP10 for specification and maintenance, including expression of NKX2-5 and NRG1. The hPSC-derived cardiomyocytes likewise responded to endocardial-secreted NRG1, adopting a trabecular fate complete with BMP10 expression. These NRG1- and BMP10-mediated *in vitro* interactions between the cardiomyocytes and endocardial cells are similar to those that occur in the developing heart.

An intricate and interdependent relationship between endocardial and myocardial cells during *in vivo* differentiation has been recently highlighted in zebrafish. In fish, Hedgehog (Hh) signaling is required for development of the myocardium (47). Hh signaling, along with vascular endothelial growth factor (Vegf) and Notch pathways, also has roles in vascular endothelial differentiation, migration, and branching (48). However, only Hh has a role in early endocardial differentiation (49). Genetic or pharmacological inhibition of Hh results in near loss of the endocardial marker, nfatc1, while leaving the differentiation of vascular endothelial populations unperturbed. Thus, Hh signaling may be of paramount importance specifically for endocardial development. Consistent with the zebrafish data, decreased Vegfa in mice results in irregular vascular endothelium formation, while the endocardium appears normal (50). These data suggest that, while Vegfa is required for general vascular development, it is not required for early endocardial morphogenesis. Additional work using myocardial-deficient *hand2* mutant zebrafish embryos revealed an endocardial-specific defect in differentiation (51, 52). Moreover, in the aftermath of genetically ablating zebrafish cardiomyocytes, endocardial cells lose their identity through loss of *nfatc1* expression to become more vascular-like (52, 53). Interestingly, in the context of myocardial-cell depletion, overexpressing Bmp2b can partially rescue endocardial progenitor cell identity by increasing Nfatc1 expression. This places BMP signaling at the crux of cardiogenic differentiation (23). Altogether, these data tell us that endocardial and myocardial differentiation are inextricably linked through a definitive and reciprocal paracrine signaling program.

## Endocardial Signaling Regulating Endocardial Differentiation

### Transcription Factor Etv2

As discussed above *Etv2* is key for endocardial differentiation from the mesoderm (36, 38, 54). However, embryonic expression of Etv2 is transient (36, 55). In mice, Etv2 is expressed within the cardiac crescent as early as E7.75, by E8.5 Etv2 labels all endothelial cells, and by E10.5 its expression is limited to the dorsal aorta (56). It is the only factor identified to date that is both necessary and sufficient for establishing endothelial, endocardial, and haematopoietic cell lineages. Deletion of *Etv2* leads to embryonic lethality by E9.5 with complete loss of blood, vessels, and endocardium. In addition, Etv2 appears to also suppress myocardial differentiation as loss of Etv2 permits myocardial expansion in mouse, zebrafish, and ESC differentiation systems (54–57).

Within the endocardium, Nkx2-5 regulates Etv2 (36) and, within the mesodermal lineage, Mesp1 is an upstream Etv2 regulator (54, 57). Downstream targets of Etv2 in the mouse include endothelial genes such as *Pecam1, Tie2/Tek*, and *VE-Cadherin/Cdh5*. Etv2 also regulates a cadre of genes involved in the fate of mesodermal progenitor cells, among them are *Gata1, Gata2, Flk1, Lmo2*, and *Scl*. All told, these studies demonstrate a reliance on Etv2 for correct endocardial specification.

### The Tyrosine Kinase Receptor VEGFR2

Vascular endothelial growth factor receptor 2 (VEGFR2/Flk1) is found within all endothelial cells at an early stage, including the endocardial precursors and endocardium (although later than the *cloche* gene in zebrafish, below). Similar to Etv2, global or conditional ablation of *Vegfr2* in mice with *Mesp1Cre* (mesodermal expression) or *Tie2Cre* (endothelial expression) leads to embryonic demise by E9.5 or E10.5, respectively, with absence of cardiac trabeculation, endocardium, blood vessels, and blood cells (29, 58). Vegfr2 may be upstream of Etv2 during early mesodermal differentiation, as overexpression of Etv2 in a *Vegfr2–/–* ESC line rescued haemato-endothelial defects (59). Moreover, in *Vegfr2* mutant embryos, Etv2 expression is significantly downregulated whereas loss of *Etv2* only slightly affected expression of Vegfr2 (60, 61). Thus, Vegfr2 plays a pivotal role in endocardial specification. It must be noted, however, that Vegfa produced by the myocardium is the primary ligand for Vegfr2 (62) and, as discussed above, Vegfa appears to be dispensable for early endocardial differentiation.

### Transcription Factor Npas4l (*Cloche)*

*Cloche* is perhaps the first gene to be identified as essential for endocardial cell development. As with the *Vegfr2* mutant mouse, the well-known *cloche* mutant zebrafish embryo also lacks the inner endocardial tube, the myocardium fails to mature, and the embryo subsequently dies whereas the endothelium of the dorsal aortae and cardinal veins appears normal (63). Thus, cloche has a dual function as a regulator of haematopoeisis and in the formation of the endocardium. There has been longstanding speculation that cloche might function upstream of *etsrp*, the zebrafish homolog to mammalian ETV2. In zebrafish, *etsrp* is downregulated in the *cloche* mutants (64) and overexpression of *etsrp* rescued the vascular defects in the *cloche* mutants (65). Over two decades later, (66) reported that the mutated gene in the *cloche* model is the transcription factor *npas4-like* (*npas4l*). Because *npas4l* is missing in mammals, based on phylogenetic analysis, and mice deficient for *Npas4* survive into adulthood without apparent haemato-vascular defects (67), an exciting area for future studies is to interrogate if there is an as-of-yet-unknown equivalent of *npas4l* in mammals, or if there is functional redundancy involving known NPAS proteins.

## Endocardial-Myocardial Crosstalk During Trabeculation

Myocardial trabeculation is a unique and life-essential morphological landmark of ventricular chamber development wherein cardiomyocytes proliferate, differentiate, and form a network of protrusions extending into the lumen of the heart. Trabeculae increase cardiac muscle mass and, prior to coronary vascularization, they permit the cardiomyocytes access to oxygen and nutrients (68). To date, all mouse models that have stunted trabeculation have a hypoplastic left ventricular wall and fail to survive past midgestation (E14.5) (9, 10). It therefore appears that trabeculation is necessary to sustain embryonic life and failure in the process precludes ventricular compaction. Indeed, this is substantiated clinically, as there are no reports of human cardiomyopathies caused by a lack of trabeculation. Mouse models have provided insight into inter-cellular communication necessary for trabeculation. These studies show that trabeculation is contingent on the endocardium, myocardium, and cardiac extracellular matrix (ECM) for proper endocardial and myocardial proliferation and differentiation (69, 70).

### Process of Trabeculation

**Figure 2:**
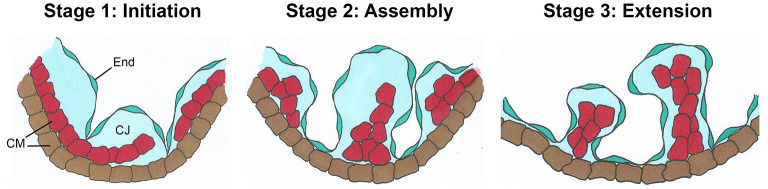
*Schematic view of the three basic stages of trabeculation*. Initiation (Stage 1) involves delamination of the innermost layer of cardiomyocytes (CMs, red) into the lumen where they form sheet-like protrusions called myocardial lamina. The endocardium (End) sends out angiogenic extensions, or sprouts, that penetrate the cardiac jelly (CJ) to directly touchdown onto the outer mycardial layer (brown). During Assembly, (Stage 2), endocardial sprouts first extend laterally underneath the myocardial lamina. Later they will assemble into individual short trabecular clusters within bubbles of cardiac jelly. Finally, in Stage 3, long sheet-like trabecular structures are formed by Extension [adapted from Qu et al. (72)].

Myocardial trabeculation is generally thought to start after cardiac looping at E9.0 in mice (6, 20, 70), although some have suggested that the process might begin as early as E8.0, when the linear heart tube is forming (71), or shortly after, at E8.5 (1). Based on recent published works (71, 72), trabeculation can be described in three steps characterized by distinct anatomical changes (Figure 2). At Stage 1 (Initiation), the inner layer of cardiomyocytes delaminates and a network of protrusions extend into the lumen, creating the myocardial lamina. The endocardium sends out “sprouts” or angiogenic extensions, penetrating the cardiac jelly to make contact or “touchdown” points with the outer layer of myocardium. At Stage 2, Assembly occurs, when endocardial sprouts move laterally under the myocardial lamina and assemble into short trabecular clusters within ECM bubbles. At the Extension Stage, Stage 3, long sheet-like trabeculae are formed within the ventricular lumen *via* cardiomyocyte proliferation inside the ECM bubble. Between E9.5 and E13.5, trabeculae rapidly grow and expand, then trabecular growth subsides with subsequent remodeling or “compaction” around E14.5 (73). Because myocardium and trabeculation initiation were evident in mouse embryos deficient of *Etv2* or *Vegfr2*, both of which lack an endocardial-lined heart tube, early myocardial differentiation may not completely depend on endocardial cells. However, endocardial cells are a prerequisite for trabecular assembly and extension, myocardial maturation, normal function, and survival.

### Endocardial-Myocardial Signaling Pathways During Trabeculation

#### Notch Signaling

One of the most thoroughly investigated signaling axes in cardiac trabeculation is the Notch pathway. At the initiation of trabeculation, there is accentuated activation of the Notch1 receptor (N1ICD) within the endocardium toward the bottom of the emerging trabeculae (6). When the Notch1 receptor or its effector RBPJk are inactivated globally or in an endothelial-specific manner, it disrupts the differentiation of trabecular endocardial and myocardial cells, reduces cardiomyocyte proliferation within the ventricles, leads to impaired trabeculation and embryonic demise at E10.5 (6, 9). Conversely, augmenting Notch1 expression through suppression of Numb (74) or Fkbp1a (75) leads to ventricular hypertrabeculation. During trabeculation, endocardial Fkbp1a (a peptidyl-prolyl isomerase) is necessary for regulating the stability of N1ICD to modulate chamber morphogenesis (75). The Notch1 ligand Dll4 is expressed within the endocardium with enriched expressed toward the base of the developing trabeculae (6, 9), whereas the Jag1 ligand is expressed in the myocardial layer (9, 76, 77). While endocardial-specific inactivation of Dll4 disrupts trabeculation, Jag1 is dispensable during the early phases of trabeculation, although it is crucial for compaction (9).

Dampening Notch signaling leads to misregulation of three key trabecular signaling cascades: Bone morphogenetic protein 10 (Bmp10), Neuregulin 1(Nrg1)-ErbB2/4, and EphrinB2/EphB4. Trabecular myocardium is enriched for Bmp10 between E9.0 and E13.5. Loss of *Bmp10* in mice results in embryonic lethality around E10.5, due to improper trabeculation and hypoplastic ventricular walls (78). It is likely that endocardial Notch is a key modulator of cardiomyocyte proliferation as (1) deletion of *RBPJk* or *Notch1* from the endocardium results in reduced myocardial Bmp10 and (2) incubating the *RBPJk* mutants in Bmp10-conditioned media rescues the phenotype (6). The second pathway, Nrg1- ErbB2/4, is initiated by release of Nrg1 from endocardial cells and acts in a paracrine fashion on myocardial tyrosine kinase receptor ErbB4 and its coreceptor ErbB2. Mice null for *Nrg1* or either ErbB receptor die between E10.5 and E11.5, with hypoplastic ventricle walls devoid of normal trabeculae (79–83). Similarly, loss of *Efnb2*, which encodes EphrinB2, and the EphB4 coreceptor causes embryonic lethality between E10.5 and E11.0, with lack of ventricular trabeculation (84). In *Notch* mutants, endocardial expression of Nrg1 is lost. Additionally, N1ICD-RBPJ regulates the transcription of endocardial *Efnb2*. What's more, expression analysis demonstrated that EprhinB2 is upstream of Nrg1. Lastly, the cardiomyocyte differentiation phenotype in *Rbpjk* mutants is rescued when the embryos are cultured with Nrg1 (6). In sum, the Notch1 pathway is central in the coordination of cardiomyocyte (Bmp10) and endocardial signaling (Nrg1 and EphrinB2) during cardiac trabeculation.

#### PlexinD1 Signaling

Sema3E/PlexinD1 signaling is known to regulate the growth of axons and the formation of the vasculature (85). More recently, it was shown that loss of the class-3 Sema receptor, *Plxnd1*, from the developing endothelium results in hypertrabeculation akin to that seen in patients with left ventricle noncompaction (86). Additionally, there was an excessive amount of cardiac jelly, or ECM, due to a decrease in expression of proteolytic genes. While ECM proteolytic gene expression was perturbed, Notch1 and its downstream targets were increased. Inhibition of Notch1 in the *Plxnd1* mutants partially rescued the ventricular phenotype, placing Plxnd1 upstream of Notch1 signaling. Importantly, one of PlexinD1's ligands, Semaphorin 3E (Sema3E), is also expressed in the developing heart, albeit more broadly than PlexinD1, and has functions in myocardial compaction. In all, these studies uncovered a novel and central role for endocardial PlexD1 and its ligand Sema3E as inhibitors of the Notch pathway during trabecular formation and ventricular compaction.

### Myocardial-Endocardial Signaling Pathways During Trabeculation

#### Ang1/Tie2 Signaling

Tie2/Tek is a tyrosine kinase receptor expressed in all endothelial cells, including endocardial cells. Since *Tie2* null embryos have no trabeculae (87, 88) and deficiency of the primary Tie2 agonist, *Angiopoietin-1 (Ang1)*, markedly reduces trabeculae formation (89, 90), it is anticipated that Ang1 is a myocardial-derived signal that cues the endocardial cells *via* Tie2 to promote trabeculation. However, the *Tie2*-deficient mouse embryos had concomitant extra-cardiac vascular defects that proved lethal, thus it was unclear if the lack of trabeculation was a primary defect, or secondary to the ubiquitous vascular abnormalities. To circumvent this problem, a unique endocardial-specific Cre mouse line (*Nfatc1*^*Cre*^) was used to delete *Tie2* exclusively from the endocardium (72), thus avoiding general vascular defects seen with global Tie2 attenuation. Indeed, removing Tie2 specifically from the endocardial cells causes lethality at midgestation due to a hyperplastic, but over-simplified trabecular network that contains fewer, but thicker trabeculae, as well as impaired endocardial sprouting (72). In the *Tie2* mutants, the hyperplastic trabeculae were the result of abnormally proliferative cardiomyocytes, associated with myocardial upregulation of Bmp10 and retinoic acid signaling, along with Erk1/2 hyperphosphorylation. Intriguingly, only the myocardial phenotypes could be partially rescued by using a pan-retinoic acid inhibitor BMS493 *in utero*. This pinpoints a role for endocardial Tie2 signaling during ventricular chamber formation which has a direct impact on trabeculation *via* paracrine suppression of retinoic acid signaling and proliferation in trabecular cardiomyocytes (72).

#### Vegf/Vegfr2 Signaling

Vegfa, the ligand for Vegfr2, is produced by the myocardium within the developing heart (62, 91, 92) and is requisite for developmental vasculo- and angio-genesis (93, 94). Global ablation of *Vegfr2* or *Vegf* haploinsufficiency in mice leads to early embryonic demise (at E9.5) caused by cardiovascular defects including loss of trabecular formation (58) (Table 1). Thus, it is widely believed that early heart development relies on a delicate control of Vegf concentration and that Vegf/Vegfr2 signaling is critical for trabeculation (3, 20, 71). A recent elegant work in mice showing that Vegfa, Vegfr2, pAKT, and Dll4 were all upregulated in the *Tie2*^*Cre*^*;Notch1*^*fl*/*fl*^ embryonic heart emphasizes the importance of the Notch pathway in restriction of Vegfa–Vegfr2 signaling during trabeculation (71). However, because overall inactivation or overexpression of Vegfa always results in severe angiogenic (especially coronary) defects prior to early embryonic demise, it is unclear whether trabeculation defects are a consequence of myocardial Vegfa deficiency or secondary to the angiogenic defect (Table 1). It is noteworthy that cardiomyocyte-specific deletion of *Vegfa* with myosin light chain 2v (MLC2v)-Cre (91) or *Tnnt2-Cre* (97) led to no apparent trabeculation defects, although the mutants died by E15.5 due to defective coronary angiogenesis and artery formation. In line with this, endocardial-specific deletion of *Vegfr2* with *Nfatc1*^*Cre*^ did not cause any apparent trabeculation defects either, although most null embryos died from E16.5 to E18.5 with defects in coronary angiogenesis, myocardial vessel formation, and thin myocardial walls (97). Thus, the specific role of Vegf/Vegfr2, as well as the potential roles of other Vegf family members, in cardiac trabeculation have not been established yet.

**Table 1:** Vegf-a mutant models and their phenotypes.

**Vegf mutants**	**Target tissue**	**Lethality**	**Angiogenic phenotype**	**Cardiac phenotype**	**Reference**
Loss of a single *VEGF-A* allele	Global	E9.5 or E11–E12	Severe defects in angiogenesis and blood island formation	Absence of trabeculation	(93, 94)
a *Vegf* hypomorphic allele *Vegf^*lo*^*	A *lacZ* knock-in into the 3′untranslated region of the mouse *Vegf* gene	Homozygous embryonic lethality by E9.0	Severely defective formation of blood islands in the yolk sac and the development of the dorsal aortae	Development of the heart is delayed; however, the endocardium is formed at E8.5–E9.0	(50)
VEGF ^120/120^ mice	Global deletion of VEGF-A_164_ and VEGF-A_188_ isoforms, expressing exclusively the VEGF_120_ isoform	About half neonates died within a few hours after birth	Bleeding in several organs	Impaired postnatal myocardial angiogenesis, resulting in ischemic cardiomyopathy	(95)
Deletion of VEGF-A *via collagen2a1-Cre*	Non-cartilagenous cell types including myocardium, in addition to chondrogenic tissues	Around E10.5 in the heterozygous state. A small percentage survive until E17.5.	Aberrant development of the dorsal aorta and intersomitic blood vessels	The endocardium appeared detached from the underlying myocardium, which was much thinner with less-developed trabeculae	(96)
CM-specific deletion of VEGF-A *via MLC2v-Cre*	Ventricular cardiac myocytes	40% died by E15.5; liveborn mutants appeared healthy	Reduced coronary microvessels	Thinned ventricular walls, depressed basal contractile function	(91)
CM-specific deletion of VEGF-A *via Tnnt2-Cre*	The myocardium	E15.5	Defective coronary angiogenesis and artery formation	Thin ventricular walls, cardiac hemorrhages, and ruptured septa, but trabeculation	(97)
Threefold overexpression of VEGF-A	Knockin at its endogenous locus	E12.5–E14.5	Aberrant coronary development	Ventricular noncompaction	(92)

### Endocardial-ECM-Myocardial Signaling Pathways During Trabeculation

Another critical player in trabeculation and compaction is the ECM, which has been well-described (20, 71). The relatively thick layer of matrix, also called cardiac jelly, can be found residing between the myocardial and endocardial cells after the heart tube forms. Trabecular initiation and maturation rely on the ECM. One fundamental function of the ECM is to promote cardiomyocyte proliferation as the myocardial mass is increased through trabeculation. Then, as trabeculation proceeds, the ECM is gradually attenuated as the endocardium touches down to make contact with the outer myocardial layer. As the cardiac jelly degrades, trabecular cardiomyocyte proliferation declines. The endocardium and myocardium have co-control over the creation and degradation of the matrix, *via* dynamic ECM remodeling processes. For instance, an elegant study showed that the transcriptional regulator Brg1, a component of the SWI/SNF ATP-dependent chromatin remodeling complex, is essential for endocardial-dependent modulation of trabecular ECM (98). Endocardial Brg1 represses a matrix metalloprotease called Adamts1; loss of Brg1 results in unfettered Adamts1 activity in the cardiac ECM where Adamts1 then prematurely degrades the ECM, stunting trabeculation. Additionally, recent genetic studies in mice have identified a role of the cerebral cavernous malformation (CCM) pathway in endocardial regulation of ECM and trabecular growth (99). Endocardial loss of each component of the CCM signaling pathway, *Krit1, Ccm2*, or *Pdcd10 via* Nfatc1Cre results in embryonic lethality due to reduced cardiac jelly and myocardial growth. The CCM phenotype is caused by inappropriately increased expression of the transcription factors Klf2 and Klf4 within the endocardium, as well as increased activity of ECM-degrading matrix metalloproteases Adamts4 and Adamts5. Another key player in embryonic endocardial regulation of ECM is a cellular importer of zinc called Solute carrier family 39 member 8 (Slc39a8, also known as ZIP8). Unlike the models described above, *Slc39a8*-null mice exhibit phenotypes consistent with decreased Adamts function resulting in impaired degradation of the ECM (100).

The well-studied Notch1 signaling pathway has recently been shown to regulate not only endocardial sprouting but also ECM degradation (71). During trabeculation, there is an interplay between the Notch1 and Nrg1–Erbb2/Erbb4 signaling cascades that is critical for balancing proper ECM deposition and degradation. For instance, Notch1 facilitates ECM degradation to enable initiation of trabeculae throughout the ventricle chambers as the endocardium sprouts touchdown on the outer myocardial layer. Nrg1, on the other hand, works to boost myocardial ECM production for trabecular assembly and extension. These data support the concept of the endocardial layer as being central to trabeculation through its regulation of the ECM and cardiomyocyte proliferation.

## Endocardial Growth During Trabeculation

During cardiac trabeculation, endocardial and myocardial cells support and interact with each other to stimulate cardiomyocyte proliferation and endocardial growth. Disrupting this endocardium-myocardium communication results in embryonic lethality due to failed cardiac growth (5, 70). Even as studies continue to highlight the essential roles of the endocardium, the regulatory mechanisms involved in endocardial proliferation and development remain largely unknown. Historically, research has focused on the transcriptional regulation of myocardial differentiation in the early stages of cardiac development. Less is known about factors that regulate endocardial ontogeny during heart development.

**Figure 3:**
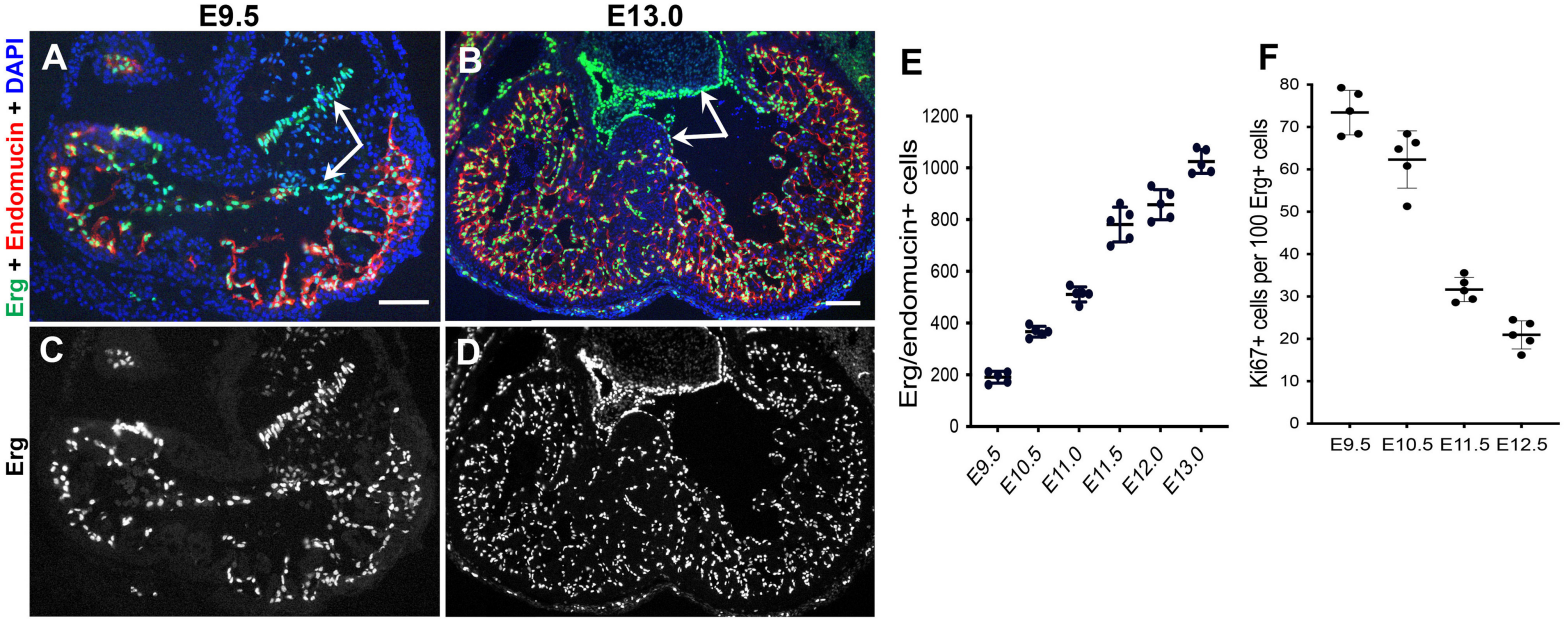
Rapid expansion of the endocardium during trabeculation. **(A–D)** Dual immunostaining of wildtype mouse heart sections for the endothelial-specific ETS transcription factor Erg (green) and the endothelial marker endomucin (red) at E9.5 and E13.0. The endocardial cells in the ventricles display high expression of endomucin, but a subset of endocardial cells undergoing EndoMT to form the mesenchymal cells of the cardiac valves are negative for endomucin (arrows). Scale bars, 100 μm. **(E)** Quantification of the total number of endocardial cells (Erg+/endomucin+ cells) indicates a rapid expansion of endocardial cells from E9.5–E13.0 during trabeculation. **(F)** However, quantification of endocardial proliferation (Ki67/Erg-positive cells) after dual immunostaining of wildtype heart sections for Ki67 and Erg at E9.5–E12.5 shows a decrease in relative endocardial cell proliferation as trabeculation progresses.

As discussed above, significant myocardial trabeculae growth begins after cardiac looping at E9.0, with rapid growth and expansion of the trabeculae from E9.5 to E13.5. Concomitantly, once the endocardial cells are specified, they must quickly proliferate to keep pace with the trabecular myocardium, leading to an expansion of endocardial cells near the end of trabeculation around E13.5 (Figure 3). Interestingly, despite the observation that the early endocardium uniquely expresses Nfatc1, the transcription factor is not necessary for regulating the specification or proliferation of endocardial cells, although it is later necessary for the formation of cardiac valves (22, 101, 102). For the most part, regulation of endocardial proliferation is uncharacterized.

Tie2 has dual roles in regulation myocardial proliferation in a paracrine manner, as discussed above, and it has a central autocrine effect on endocardial growth and proliferation that lays the foundation for normal trabeculation. Tie2 is dispensable for pan-endothelial differentiation and the first stages of vascular assembly (103), but it is required for endocardial sprout formation and growth. Utilizing the same endocardial-specific *Nfatc1*^*Cre*^ line to conditionally remove Tie2 from the endocardium as described above, Qu et al. (72) demonstrated that Tie2 signaling is a prerequisite to trabeculation. Loss of endocardial Tie2 results in impaired embryonic endocardial sprouting and growth as early as E9.0 due to decreased endocardial cell proliferation and impaired migration. Thus, Tie2 promotes endocardial proliferation and development of the angiogenic extensions, or sprouts, involved in initial stages of trabeculation. Interestingly, endocardial loss of *Tie2* interfered with the endocardial gene signature, with decreased expression of *Sox7, Arap3* and *Tmem100* (72). Global knockout of these genes in mice leads to *in utero* demise with impaired trabeculation involving disturbed endocardial network formation. However, the cardiac defects are likely secondary, because when these genes are deleted specifically from the endocardium using *Nfatc1*^*Cre*^, all lines display a normal endocardial network and proper trabeculation (72, 104). Therefore, more studies are needed to better understand how Tie2-Ang1 signaling regulates the endocardial proliferation necessary to support trabeculation.

As discussed above, Etv2 and Vegfr2 are both specifically expressed in endothelial cells and are critical for their specification. Loss of either gene results in embryonic mortality with global depletion of all endothelial cells, so it is plausible to argue that both are required for endothelial and endocardial growth. However, conditional knockout mice of *Etv2* with *Tie2*^*Cre*^ (active in the endothelial lineage) or *Nfatc1*^*Cre*^ (active in endocardial cells from E9.0, about 1 day later than *Tie2*^*Cre*^) (71) were healthy (72, 105). This is consistent with the observation that Etv2 has transient endocardial/endothelial expression from E7.75-E9.5 (36). These results support a role for Etv2 during a restricted developmental window, when it activates haematopoietic and endothelial differentiation (54). Similarly, endocardial-specific deletion of *Vegfr2* with *Nfatc1*^*Cre*^ also led to no apparent endocardial defects, although most null embryos died from E16.5 to E18.5 with abnormalities in coronary angiogenesis and myocardial vessel formation (97).

Other studies emphasize the importance of biomechanical cues in regulating endothelial proliferation, specifically during later stages of ventricular chamber morphogenesis. A recent report using a zebrafish model showed that endocardial cell proliferation during the endocardial ballooning phase and chamber morphogenesis is not controlled by Vegf signaling, rather it depends on hemodynamics and myocardial-derived Bmp (53). Bornhorst et al. (106) also used zebrafish to further investigate the biomechanics involved in cardiogenic regulation. They demonstrated that the expansion of the myocardium creates tension on the endocardium, where the biomechanical transducer VE-Cadherin responds by upregulating endocardial proliferation *via* Yap1 nuclear translocation. Taken together, these studies suggest that endocardial specification is Etv2- and Vegfr2- dependent, whereas the subsequent endocardial growth and expansion during trabeculation are not.

Lastly, it is evident that the Notch1-Dll4 signaling pathway is required for endocardial growth during trabeculation, as endocardial-specific deletion of *Notch1* or its ligand, *Dll4*, with *Nfatc1*^*Cre*^ leads to impaired endocardial sprouting and a simplified endocardial network (9, 71). However, additional studies are required for further confirmation and to determine how Notch1-Dll4 signaling modulates endocardial proliferation during the critical trabeculation timeframe. Moreover, potential interactions between Notch1-Dll4 and Tie2-Ang1 signaling pathways remain to be explored.

## Conclusion

The endocardium and myocardium interact through dynamic paracrine signaling pathways in all stages of cardiac development. Endocardial cells secrete signaling mediators such as Nrg1 that modulate cardiomyocyte development and survival. Reciprocally, cardiomyocytes promote endocardial cell proliferation, assembly, and survival through Angiopoietin-1. In this review, we focus on these critical reciprocal exchanges between the myocardium and endocardium in early cardiac development, including the origins of the endocardial and myocardial lineages, endocardial and myocardial crosstalk during differentiation and trabeculation, and signaling essential for endocardial growth during trabeculation. While much attention has been focused on the transcriptional regulation of myocardial development during early cardiogenesis, with the role of the endocardium being relegated to the process of EMT during valvulogenesis (11), it is now quite clear that the endocardium plays a central role in choreographing most of the major morphological processes required for ventricular chamber formation. Specification of the cardiac lineages as well as the cellular proliferation and differentiation necessary for valve and chamber morphogenesis, all rely on a complex interplay between multiple pathways such as Notch, Wnt, Bmp, Nrg1-ErbB2/4, and EphrinB2/EphB4. Endocardial Tie2 plays complimentary roles during ventricular chamber trabeculation by promoting endocardial proliferation and sprouting, while preventing myocardial hypertrabeculation through suppression of retinoic acid signaling within cardiomyocytes. Beyond this, endocardial cells have roles in coronary vascular development and in haematopoiesis, which suggests that they have roles during heart regeneration and could provide novel progenitors for mural cells of the heart (10, 107). Thus, the endocardium is a promising target for therapeutic intervention both to maintain homeostasis within the heart and to stimulate cardiomyocyte replenishment during cardiac disease or injury.

## Author Contributions

XQ and HB wrote the manuscript and generated the figures. XQ, HB, and CH revised the manuscript. All authors contributed to the article and approved the submitted version.

## Funding

We are grateful for funding support from National Institutes of Health HL R01 DK125895-01.

## Conflict of Interest

The authors declare that the research was conducted in the absence of any commercial or financial relationships that could be construed as a potential conflict of interest.

## Publisher's Note

All claims expressed in this article are solely those of the authors and do not necessarily represent those of their affiliated organizations, or those of the publisher, the editors and the reviewers. Any product that may be evaluated in this article, or claim that may be made by its manufacturer, is not guaranteed or endorsed by the publisher.
